# Binary-state speciation and extinction method is conditionally robust to realistic violations of its assumptions

**DOI:** 10.1186/s12862-018-1174-5

**Published:** 2018-05-08

**Authors:** Andrew G. Simpson, Peter J. Wagner, Scott L. Wing, Charles B. Fenster

**Affiliations:** 1 Department of Paleobiology, National Museum of Natural History, Smithsonian Institution, Washington D.C., USA; 2 Department of Earth & Atmospheric Sciences and School of Biological Sciences, University of Nebraska, Lincoln, USA; 3 Department of Biology and Microbiology, South Dakota State University, Brookings, USA; 4 Program in Behavior, Ecology, Evolution, and Systematics, University of Maryland, College Park, USA

**Keywords:** Character evolution, Systematics, BiSSE, Simulated evolution

## Abstract

**Background:**

Phylogenetic comparative methods allow us to test evolutionary hypotheses without the benefit of an extensive fossil record. These methods, however, make simplifying assumptions, among them that clades are always increasing or stable in diversity, an assumption we know to be false. This study simulates hypothetical clades to test whether the Binary State Speciation and Extinction (BiSSE) method can be used to correctly detect relative differences in diversification rate between ancestral and derived character states even as net diversification rates are declining overall. We simulate clades with declining but positive diversification rates, as well those in which speciation rates decline below extinction rates so that they are losing richness for part of their history. We run these analyses both with simulated symmetric and asymmetric speciation rates to test whether BiSSE can be used to detect them correctly.

**Results:**

For simulations with a neutral character, the fit for a BiSSE model with a neutral character is better than alternative models so long as net diversification rates remain positive. Once net diversification rates become negative, the BiSSE model with the greatest likelihood often has a non-neutral character, even though there is no such character in the simulation. BiSSE’s usefulness in detecting real asymmetry in speciation rates improves with clade age, even well after net diversification rates have become negative.

**Conclusions:**

BiSSE is most useful in analyzing clades of intermediate age, before they have reached peak diversity and gone into decline. After this point, users of BiSSE risk incorrectly inferring differential evolutionary rates when none exist. Fortunately, most studies using BiSSE and similar models focus on rapid, recent diversifications, and are less likely to encounter the biases BiSSE models are subject to for older clades. For extant groups that were once more diverse than now, however, caution should be taken in inferring past diversification patterns without fossil data.

**Electronic supplementary material:**

The online version of this article (10.1186/s12862-018-1174-5) contains supplementary material, which is available to authorized users.

## Background

A key recurring question in evolutionary biology is what evolutionary novelties lead to shifts in speciation and/or extinction rates. Historically, the fossil record has been used to address this question. More recently, developments in phylogenetic comparative methods present an alternative to use for groups that have poor to nonexistent fossil records [1]. Among these developments are probabilistic models that enable researchers to infer both the evolution of the character traits responsible for diversification patterns as well as the diversification rates themselves [2, 3]. Phylogenetic comparative methods have now been directed at a variety of different taxonomic groups (e.g. flowering plants [4]; mammals [5]; birds [6]).

A limitation of phylogenetic methods is that phylogenetic trees represent only the ancestors of termini represented in the phylogeny. Reconstructing the richness of unsampled subclades is extremely difficult. An important assumption of many phylogenetic methods is that they require ultrametric trees and thus require all termini to represent extant taxa, with the result that extinct lineages within a study group are necessarily left unsampled and so become problematic [7–9]. In theory, entirely neontological datasets could be used to reconstruct histories of declining clades because the likelihood functions for net-negative diversification rates are well-defined. However, neontological studies from real clades that are in decline often fail to identify the net-negative diversification rates these lineages experienced in the past [8]. Many extant groups were more diverse in the past, including lineages of invertebrates (e.g. brachiopods, bryozoans, crinoids [10]), vertebrates (gar fish, crurotarsans, hominids [11]), and plants (lycopods, cupressaceous conifers, gnetophytes, sycamores [12]). The ubiquity of declining lineages suggests that such decline is a general behavior of clades, with diversifying lineages merely having yet to reach the declining phase of their history [13].

Multiple studies have examined the effects that changing diversification rates have on the usefulness of phylogenetic comparative methods in reconstructing relationships between state transitions and diversification [14, 15]. Some neontological approaches enable use of phylogenetic methods that account for some of these effects (e.g. [16] for successively-diversifying subclades, [17] for density-dependent diversification). Here we examine mechanisms that limit the usefulness of phylogenetic methods to correctly infer relationships between the character states and diversification.

In this study, we set out to address three questions concerning the behavior of the binary state speciation and extinction (BiSSE) method [2], the simplest of a family of similar (SSE) methods that use a likelihood optimization routine to compare models of speciation, extinction, and state transition given a phylogenetic tree of extant taxa. Here we use BiSSE to analyze a single character with two possible states, with speciation and extinction rates associated with each state. Although SSE methods account for diversification rate shifts as a result of changes between the character states, persistently declining diversification rates still violate the assumptions of all SSE methods. Our three questions are as follows: (1) how severely must BiSSE’s assumptions of constant speciation and extinction rates be violated before it fails to be useful when modeling the effects of the character states on diversification parameters? Under the condition where the character states have no effect on speciation rate we predicted the most likely BiSSE models would be those with higher rates of speciation in a clade’s ancestral state. This is because the frequency of a character’s ancestral state must necessarily be high early in a clade’s history, since the derived state has not had a chance to evolve yet. By the time the derived state has become common, speciation rate has declined. For this reason, the most likely BiSSE model would have the derived state associated with the decline in speciation rates, even though it is not. (2) Is BiSSE’s power to detect biases in transition rates between the character states compromised if characters evolve in a punctuated fashion (sensu [18]; see also ClaSSE, [19])? We predicted that a punctuated equilibrium simulation would result in the best BiSSE model featuring the derived state having a lower rate of reversion to the ancestral state than vice-versa. The rationale is that an asymmetry in favor of the ancestral state transitioning to the derived state reflects events early in clade history when the ancestral state is common and the clade is diversifying rapidly. (3) Do decaying, constant, or increasing extinction rates also affect how useful BiSSE is for recovering speciation rate? Using reasoning similar to the first question we predicted that decreasing extinction rates associated with both character states would result in the most likely BiSSE model having the ancestral state associated with higher extinction rates.

## Results

### Evolutionary history reconstruction for continuous-time evolution of a neutral trait

**Fig. 1:**
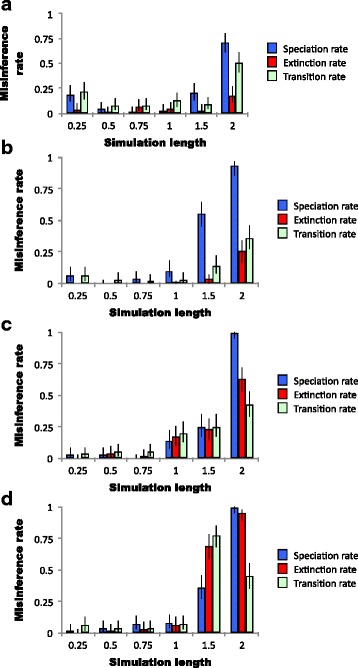
Risk of misinterpreting BiSSE results to incorrectly infer evolutionary rate asymmetry on simulated trees when no asymmetry exists. Simulation length is the amount of time the simulation ran to generate the simulated clade, with one unit being roughly the time necessary for the λ to drop below the μ (see Fig. 3). **a**, **b** λ and μ both decline, but at different rates such that μ eventually overtakes λ, as in Fig. 3a. **c** λ declines while μ is constant, so that eventually extinction dominates, as in Fig. 3b. **d** λ decreases while μ increases, as in Fig. 3c. The relative rates of speciation and extinction are the same as those described in the corresponding graph in Fig. 3. **a** Character evolution follows a punctuated equilibrium simulation, whereby character evolution only occurs during speciation events. **b**, **c**, **d** Character evolution is continuous with time and proceeds irrespective of speciation. Misinference rate represents the results of an AIC-based likelihood ratio test on BiSSE model fits in which the character states had or did not have an effect on $\widehat{\uplambda}$, $\widehat{\upmu}$, or $\widehat{\mathrm{q}}$, respectively. Each combination of parameters and simulation length represents 100 simulated trees. The blue bars represent differences in $\widehat{\uplambda}$, the red bars $\widehat{\upmu}$, and the green bars $\widehat{\mathrm{q}}$. Error bars represent the 95% confidence limits on the actual rate of misinference based on the size of the sample. In these simulations, the true evolutionary rates associated with ancestral and derived states are equal. Abbreviations of rates and symbols are in Table 5

For simulations of a neutral character for length less than one time unit, the BiSSE model with the highest likelihood rarely includes a non-neutral character (Table 1, Fig. 1b-d). For simulation lengths greater than one, however, the best model frequently does have a non-neutral character. The simulation length threshold after which the most likely BiSSE model has the character being non-neutral does depend on whether μ is changing as well as λ, or if μ is constant and only λ is time-variant.

**Table 1:** Proportions of simulations in which the most likely BiSSE model included character states affecting evolutionary rate when no such effect existed.

Simulation model	Simulation length	λ	μ	q
Punc. Eq., μ decreasing	0.25	0.19^***^	0.04	0.22^***^
	0.5	0.05	0.02	0.08
	0.75	0.02	0.07	0.08
	1	0.03	0.05	0.13^*^
	1.5	0.21^***^	0.03	0.09
	2	0.71^***^	0.18^***^	0.51^***^
Continuous, μ decreasing	0.25	0.06	0	0.06
	0.5	0	0	0.03
	0.75	0.04	0	0.02
	1	0.1	0.01	0.03
	1.5	0.55	0.04	0.14^**^
	2	0.93^***^	0.26^***^	0.36^***^
Continuous, μ constant	0.25	0.03	0	0.04
	0.5	0.03	0.04	0.05
	0.75	0	0.02	0.05
	1	0.14^**^	0.17^***^	0.2^***^
	1.5	0.25^***^	0.23^***^	0.25^***^
	2	0.99^***^	0.63^***^	0.43^***^
Continuous, μ increasing	0.25	0.02	0	0.06
	0.5	0.04	0.02	0.04
	0.75	0.07	0.03	0.04
	1	0.08	0.06	0.07
	1.5	0.36^***^	0.69^***^	0.77^***^
	2	0.99^***^	0.95^***^	0.45^***^

The simulation model specifies the parameters we used to simulate the clade. The first (“Punc. Eq.”) set used a punctuated equilibrium simulation of character evolution wherein transitions between character states occur only during speciation events. The rest (“Continuous”) simulate character evolution wherein state changes are independent of speciation events. Our first two sets have both λ and μ declining with time as in Fig. 3a. The third has μ constant and only λ declining as in Fig. 3b. The final set has μ increasing as in Fig. 3c. Simulation length refers to the amount of simulated time that the program was run; one unit represents approximately the time needed for extinction to outpace origination (see Fig. 3). λ, μ, and q represent the rates (out of 100 runs) for a likelihood ratio test producing a statistically significant difference between the BiSSE models in which the character states affected the corresponding evolutionary rate versus those in which the character states had no effect. The significance threshold for our likelihood ratio test is α = 0.05. The number of asterisks indicates whether the rate at which the most likely BiSSE models include evolutionary rate asymmetry statistically exceeds than the expected rate following a sequential Bonferroni correction (+ *P* < 0.1, ^*^ *P* < 0.05, ^**^ *P* < 0.01, ^***^ *P* < 0.001). Abbreviations of λ, μ, and q are explained in Table 5

When the most likely BiSSE model allows $\widehat{\uplambda}$_0_ ≠ $\widehat{\uplambda}$_1_, this asymmetry is not always $\widehat{\uplambda}$_0_ > $\widehat{\uplambda}$_1_ (Table 2). For simulations in which the best model includes speciation rate asymmetry, Table 3 shows only those runs on which the likelihood ratio test identified the difference between the model in which $\widehat{\uplambda}$_0_ ≠ $\widehat{\uplambda}$_1_ is better than the model in which $\widehat{\uplambda}$_0_ = $\widehat{\uplambda}$_1_ is statistically significant at the 0.05 level. Excluding simulations for which the difference between models is not significant, $\widehat{\uplambda}$_0_ > $\widehat{\uplambda}$_1_ is more common for simulations greater than length one in both our punctuated equilibrium simulation and in our constant μ simulation. The trend of $\widehat{\uplambda}$_0_ > $\widehat{\uplambda}$_1_ occurring more often with increasing clade age does not occur in our continuous time models in which μ is not constant, however (Table 3).

**Table 2:** Estimates of systematic bias in directionality of differences in evolutionary rates of most likely BiSSE models when no real (simulated) differences in evolutionary rates exist

Simulation	Length	Speciation		Extinction			Transition	
		$\widehat{\uplambda}$_0_ > $\widehat{\uplambda}$_1_	$\widehat{\uplambda}$_1_ > $\widehat{\uplambda}$_0_	$\widehat{\upmu}$_0_ > $\widehat{\upmu}$_1_	$\widehat{\upmu}$_1_ > $\widehat{\upmu}$_0_	μ = 0	$\widehat{\mathrm{q}}$01 > $\widehat{\mathrm{q}}$10	$\widehat{\mathrm{q}}$10 > $\widehat{\mathrm{q}}$01
Punc. Eq., μ decreasing	0.25	32	68	50	48	2	36	64
	0.5	45	55	52	48	0	42	58
	0.75	49	51	56	44	0	42	58
	1	54	46	47	52	1	51	49
	1.5	56	44	48	46	6	50	50
	2	60	40	15	13	72	63	37
Continuous, μ decreasing	0.25	54	46	34	56	10	59	41
	0.5	56	44	44	46	10	49	51
	0.75	48	52	52	48	0	50	50
	1	49	51	52	48	0	49	51
	1.5	49	51	46	54	0	48	52
	2	49	51	56	44	0	46	54
Continuous, μ constant	0.25	45	55	38	59	3	55	45
	0.5	49	51	43	56	1	57	43
	0.75	49	51	46	54	0	48	52
	1	47	53	50	50	0	46	54
	1.5	48	52	51	49	0	50	50
	2	60	40	60	40	0	62	38
Continuous, μ increasing	0.25	47	53	40	57	3	48	52
	0.5	40	60	43	56	1	47	53
	0.75	47	53	47	53	0	56	44
	1	51	49	51	49	0	49	51
	1.5	58	42	48	52	0	49	51
	2	46	54	46	54	0	55	45

The simulation specifies the model parameters we used to simulate the clade. The first (“Punc. Eq.”) set used a punctuated equilibrium simulation of character evolution wherein transitions between character states occur only during speciation events. The rest (“Continuous”) used a simulation of character evolution wherein state changes are independent of speciation events. Our first two sets have both speciation and extinction rates declining with time as in Fig. 3a. The third has extinction rate constant and only speciation declining as in Fig. 3b. The final set has extinction rate increasing as in Fig. 3c. Length refers to the simulated time in the run: one unit is approximately the amount of time needed for speciation rate to decay below extinction rate (see Fig. 3). λ, μ, and q refer to the parameters being estimated (Table 5) Speciation, extinction, and transition refer to the parameters being estimated. These results do not reflect whether our likelihood ratio tests found statistically significant differences between parameters, but only the sign of the difference between the estimated values

**Table 3:** Estimates of systematic bias in directionality of differences in evolutionary rates of most likely BiSSE models when no real (simulated) differences in evolutionary rates exist.

Simulation	Length	Speciation		Extinction		Transition	
		$\widehat{\uplambda}$_0_ > $\widehat{\uplambda}$_1_	$\widehat{\uplambda}$_1_ > $\widehat{\uplambda}$_0_	$\widehat{\upmu}$_0_ > $\widehat{\upmu}$_1_	$\widehat{\upmu}$_1_ > $\widehat{\upmu}$_0_	$\widehat{\mathrm{q}}$01 > $\widehat{\mathrm{q}}$10	$\widehat{\mathrm{q}}$10 > $\widehat{\mathrm{q}}$01
Punc. Eq., μ decreasing	0.25	7	12	2	7	12	2
	0.5	1	4	0	1	4	0
	0.75	1	1	5	1	1	5
	1	3	0	5	3	0	5
	1.5	10	11	2	10	11	2
	2	46	25	7	46	25	7
Continuous, μ decreasing	0.25	3	3	0	0	2	4
	0.5	0	0	0	0	1	2
	0.75	2	2	0	0	1	1
	1	4	6	0	1	1	2
	1.5	24	31	2	2	7	7
	2	43	50	17	9	12	24
Continuous, μ constant	0.25	1	2	0	0	0	4
	0.5	0	3	0	4	2	3
	0.75	0	0	2	0	0	5
	1	6	8	8	9	9	11
	1.5	16	9	15	8	10	15
	2	59	40	41	22	30	13
Continuous, μ increasing	0.25	1	1	0	0	3	3
	0.5	1	3	0	2	3	1
	0.75	3	4	1	2	2	2
	1	7	1	4	2	5	2
	1.5	21	15	35	34	38	39
	2	46	53	45	50	23	22

Unlike Table 2, this Table 3 counts only those runs in which the asymmetry in evolutionary rates was found to be statistically significant via a likelihood ratio test on the respective model fits. The simulation specifies the model parameters we used to simulate the clade. The first set used a punctuated equilibrium simulated of character evolution wherein transitions between character states occur only during speciation events. The rest used a simulated of character evolution wherein state changes are independent of speciation events. Our first two sets have both speciation and extinction rates declining with time as in Fig. 3a. The third has extinction rate constant and only speciation declining as in Fig. 3b. The final set has extinction rate increasing as in Fig. 3c. Length simulated time in the run: one unit is approximately the amount of time needed for speciation rate to decay below extinction rate (see Fig. 3). Speciation, extinction, and transition refer to the parameters being estimated (Table 5)

In those simulations in which the best model has $\widehat{\uplambda}$_0_ ≠ $\widehat{\uplambda}$_1_, this model tends to also have $\widehat{\upmu}$_0_ ≠ $\widehat{\upmu}$_1_, as well (Table 4). Of the 315 runs in which the best model had $\widehat{\upmu}$_0_ ≠ $\widehat{\upmu}$_1_, 71 did not provide a better fit for $\widehat{\uplambda}$_0_ ≠ $\widehat{\uplambda}$_1_. Of the total 1800 runs, 468 had better fit for $\widehat{\uplambda}$_0_ ≠ $\widehat{\uplambda}$_1_. The most likely BiSSE model is far more likely to include rate asymmetry in both $\widehat{\uplambda}$ and $\widehat{\upmu}$ than would be expected by chance alone (Fisher’s exact test *P* = 0.0001; Table 4).

**Table 4:** Proportions of simulations in which the most likely BiSSE model included asymmetry in $\widehat{\uplambda}$ versus simulations in which the most likely model included asymmetry in $\widehat{\upmu}$

Focal parameter	Covariate parameter	Ratio	Proportion
A.			
Asymmetry in Speciation	Asymmetry in Extinction	26/121	0.21
No Asymmetry in Speciation	Asymmetry in Extinction	13/479	0.03
Asymmetry in Extinction	Asymmetry in Speciation	26/39	0.67
No Asymmetry in Extinction	Asymmetry in Speciation	95/561	0.17
B.			
Asymmetry in Speciation	Asymmetry in Extinction	244/468	0.52
No Asymmetry in Speciation	Asymmetry in Extinction	71/1332	0.05
Asymmetry in Extinction	Asymmetry in Speciation	244/315	0.77
No Asymmetry in Extinction	Asymmetry in Speciation	224/1485	0.15

The numerator in the ratio is the number of runs in which the most likely BiSSE model includes asymmetry as described for both the focal parameter and the covariate parameter. The denominator in the ratio represents the total number of runs for which the most likely BiSSE model includes asymmetry in the focal parameter only. For instance, in the second row of (A), there were 13 runs in which the best model included the character states having an effect on $\widehat{\upmu}$ but not on $\widehat{\uplambda}$, and 479 total runs in which the best model did not include the character states having an effect on $\widehat{\upmu}$ but not on $\widehat{\uplambda}$. Proportions are the decimal values of the respective ratios. Most likely BiSSE models are more likely to include rate asymmetry in both $\widehat{\upmu}$ and $\widehat{\uplambda}$ together in the same run than expected by chance in both of our simulation models (Fisher’s Exact Test *P* < 0.0001). In all of these runs, there was no effect of the character states on either λ or μ. (A) tabulates results from a punctuated equilibrium simulation of character evolution. (B) tabulates results from a continuous-time simulation of character evolution.

The most likely BiSSE models often estimate $\widehat{\upmu}$ to be zero in a number of simulations, especially of length 0.5 and lower (Table 2). However, not a single simulated clade has more than 56% of the species generated survive. Concluding that extinction is negligible because the best model includes $\widehat{\upmu}$ = 0 would be incorrect.

### Evolutionary history reconstruction for punctuated evolution of a neutral character

In general, the results of our punctuated equilibrium simulation and our continuous evolution simulation are similar, but there are some details in which they differ. In clades created using our punctuated equilibrium simulation, the most likely BiSSE models often include non-neutral characters in both old (length > 1) and very young (length = 0.25) clades, but not for intermediate age clades (0.5 and 0.75) (Table 1, Fig. 1a). Additionally, in punctuated equilibrium simulations, the most likely model is far more likely to include $\widehat{\upmu}$ = 0 than continuous evolution simulations (Table 2).

### Reconstruction for both continuous-time and punctuated evolution of a non-neutral character

**Fig. 2:**
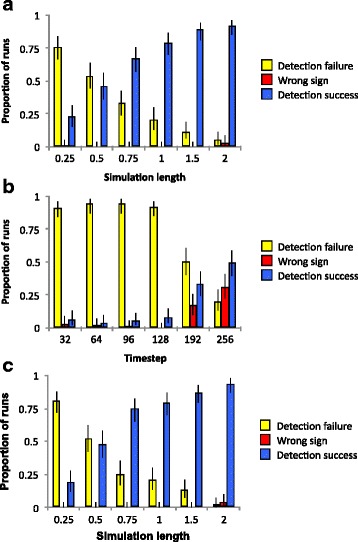
Ability of BiSSE fits to enable detection of speciation rate asymmetry on simulated trees when asymmetry does exist (not $\widehat{\upmu}$ or $\widehat{\mathrm{q}}$, unlike Fig. 1). In (**a**), λ_1_ is 1.33 times λ_0_ at any given point in time, but both decline exponentially with time as in Fig. 3a. Moreover, changes between character states occur only during speciation events, as predicted by punctuated equilibrium. In (**b**), λ_1_ is 1.05 times λ_0_, and changes between character states occur only during speciation events, as predicted by punctuated equilibrium. In (**c**), λ_1_ is 1.33 times λ_0_, but character evolution occurs independently of speciation. The yellow bar represents the proportion of runs in which the most likely BiSSE model did not include a difference in λ values, even when one existed. The red bar signifies runs in which the best model included asymmetry in $\widehat{\uplambda}$, but in the wrong direction, estimating $\widehat{\uplambda}$_0_ to be greater than $\widehat{\uplambda}$_1_. The blue bar indicates that that the best model is the most correct, incorporating the real difference between λ_0_ and λ and found a difference of the correct sign. All three sets of bars refer to estimation speciation rates. Proportion of runs represents the proportion (out of 100 runs) that each result for simulation length. Length of simulation is scaled such that one unit is approximately the time required for speciation rate to drop below extinction rate (see Fig. 3a). Error bars represent the 95% confidence limits on the actual frequency of the particular type of estimation being represented. Assessment of statistical significance is conducted using a likelihood ratio test

In all simulations where λ_0_ and λ_1_ are unequal, the most likely BiSSE model is increasingly likely to identify $\widehat{\uplambda}$_0_ ≠ $\widehat{\uplambda}$_1_ as clade age increases (Fig. 2). The most likely model also more often has $\widehat{\uplambda}$_0_ ≠ $\widehat{\uplambda}$_1_ when λ_1_/λ_0_ is 1.33 instead of 1.05. The probability that the most likely model correctly has $\widehat{\uplambda}$_1_ > $\widehat{\uplambda}$_0_ increases even as clades reach their peak richness and go into decline. We do not observe a significant difference between simulations running a continuous-time or punctuated model of character evolution.

When λ_1_/λ_0_ is 1.05, we also detect the bias observed when the simulated character is neutral: the most likely BiSSE model has $\widehat{\uplambda}$_0_ > $\widehat{\uplambda}$_1_, the opposite of what we simulated. As with the simulations with a neutral character, this effect is not detect the *p* = 0.05 level using our likelihood ratio test on length 1 simulations and shorter, but becomes observable above length 1. This effect is much stronger in simulations where λ_1_/λ_0_ = 1.05 than in those with λ_1_/λ_0_ = 1.33, with the former having 31 out of the 100 runs of length two exhibiting this type of misleading model fit, but only four out of 100 for the latter.

## Discussion

As we predicted, when applied to declining clades the most likely BiSSE model often has the character being non-neutral even when the simulated character is neutral. Although BiSSE models more often have better support for $\widehat{\uplambda}$_1_ > $\widehat{\uplambda}$_0_ (Table 2), the asymmetry of the most likely model sometimes lies in the other direction. This bias may exist because the relatively simple models that BiSSE is using cannot simulate the more complicated ways in which these rates are changing. Since both character states have become common by the end of the simulation, it is possible that the particular configuration of the tree is better approximated by a model with $\widehat{\uplambda}$_0_ > $\widehat{\uplambda}$_1_, even though BiSSE models cannot accurately approximate the processes underlying the pattern of the tree. This is a similar phenomenon to that observed by Rabosky & Goldberg [14], but in our case the ‘hidden character’ is a property of clade evolution and not a second binary character as would be modeled by HiSSE [20].

We predicted that our punctuated equilibrium simulation would demonstrate $\widehat{\mathrm{q}}$ being subject to the same biases as $\widehat{\uplambda}$. We did observe this, but we observed that $\widehat{\mathrm{q}}$ estimates are biased in our continuous-time character evolution model as well (Table 1, Fig. 1). As mentioned above, we suspect that the limited selection of models that BiSSE can fit is forcing character states to coevolve with the declining diversification rates because a model featuring changing diversification rates in any other way is not available.

Time-variance in μ has subtle effects on the overall trends we observe in this study, but we identify no clear patterns. In simulations of anagenetic character evolution of a neutral character, constant μ results in the best BiSSE model having a non-neutral character even in length-1 simulations (Table 1, Fig. 1), as clades are just reaching peak diversity. However, in the other simulations it is not until after length-1 that the most likely BiSSE models include non-neutral characters when simulated characters are neutral. In contrast, we predicted that BiSSE would be more biased with increasing μ instead of constant μ. An explanation for this behavior of BiSSE is not apparent.

We observed a number of other behaviors of BiSSE that we did not anticipate. For simulations in which the most likely BiSSE model includes asymmetry in $\widehat{\uplambda}$, it is also more likely to include asymmetry in $\widehat{\upmu}$. This is probably because the stochastic variation that creates the simulated phylogenetic trees makes for some trees that more severely violate BiSSE’s assumptions than others. More worrying is BiSSE’s tendency to fit higher likelihood scores for models featuring unequal $\widehat{\uplambda}$ and $\widehat{\upmu}$ in very short simulations that use the punctuated equilibrium model (Table 1, Fig. 1a). This is of concern for two reasons: first, punctuated equilibrium is commonly observed in fossil data [18, 21], meaning that cladogenic character evolution is a possibility that systematic biologists must be prepared for. Second, workers are likely to apply BiSSE to young, rapidly diversifying clades (e.g. nightshades [22]; color-varying birds [6]). We suggest that the reason the most likely models of young clades have asymmetrical speciation rates is because of stochastic variation: some clades get “lucky” and have an early burst of diversification that by chance is associated with one or the other character. Thus the BiSSE algorithm provides a better fit for a highly incorrect model with a non-neutral character than for a less incorrect model. Our minimum cutoff of 36 species in the punctuated equilibrium model would exacerbate this acquisition bias toward clades with such early bursts of diversification, and it is possible that we would not observe it were our cutoff lower. Unfortunately, the reasoning by which evolutionary biologists select clades of study has similar biases and problems.

In contrast to what Machac [15] observed with QuaSSE, increasing clade age does not decrease the usefulness of BiSSE’s model fitting to detect real asymmetry in λ. This is not a function of the older clades having more species, because the BiSSE model with the greatest likelihood is more likely to include asymmetry even as clades are in decline (Fig. 2). We suggest that this is because BiSSE is computationally simpler than QuaSSE, and has more statistical power to detect rate asymmetry. As expected, if the asymmetry in λ is small, the fit for models with $\widehat{\uplambda}$_0_ ≠ $\widehat{\uplambda}$_1_ is worse than when the real asymmetry is large. Moreover, in older clades the bias that causes BiSSE models to have better fits for $\widehat{\uplambda}$_0_ > $\widehat{\uplambda}$_1_ can overpower the improvement in model fit reflecting actual asymmetry in the other direction. If λ_1_/λ_0_ is large, real rate asymmetry overpowers the bias.

There is an alarming tendency for the most likely BiSSE model to have $\widehat{\upmu}$=0 for both character states. The probability of this happening is greater in longer runs or in punctuated equilibrium scenarios. These parameter estimates from the most likely BiSSE models are misleading because no more than 56% of the total species survived to the end in any of our simulations. Extinction is pervasive in the fossil record, a fact that is sometimes overlooked by studies focused on modern data (see [23] for discussion). Workers not aware of this might use BiSSE and misinterpret the results as genuinely signifying that a clade has zero extinction in its history.

We propose that BiSSE is best suited for study of clades of intermediate age, which have already undergone their initial pulse of rapid diversification but have not yet reached peak diversity. BiSSE is more likely to enable researchers to recover true rate asymmetry than constant rate estimators [15], and its ability to fit (relatively) correct models featuring true rate asymmetry improves with clade age even as assumptions of positive diversification are violated. Predictably, the larger the asymmetry in evolutionary rates, the more useful BiSSE is at detecting it. Unfortunately, in old, declining clades, as well as very young clades, the most likely BiSSE models are prone to feature rate asymmetry even if there is none. This makes clade selection an important consideration when using BiSSE.

At present, state speciation and extinction models such as BiSSE, QuaSSE, and their cousins are the only phylogenetic methods capable of assessing the effects of the character states on diversification rate as well as directionality in character evolution. Unfortunately, available SSE models are unable to make use of fossil data, generally requiring ultrametric trees. Non-SSE models such as BioGeoBEARS [24] do use fossil data for ancestral reconstructions. We suggest that future development of phylogenetic methods focus on the ability to incorporate fossil data. Presently, BiSSE and similar methods are useful tools in an evolutionary biologist’s arsenal, but to use them exclusively will lead to systematic bias in assessing evolutionary history. The ability of phylogenetic methods to accurately reconstruct past diversification patters is improving, but as we demonstrate there remain numerous situations where their use may be compromised [14, 15].

## Conclusions

BiSSE is moderately robust to the violation of the assumptions we investigate; a clade must be in decline for it to have a high probability of generating misleading results, unlike QuaSSE [15]. Furthermore, the most likely BiSSE models often reflect real asymmetries in diversification rate associated with a character but BiSSE analyses are prone to be misleading during clade decline due to violations of its assumptions. BiSSE’s robustness in recovering differences in diversification between character states increases with greater asymmetry, but fairly modest asymmetries (e.g. a factor of 1.33) can still be detected reliably, even in clades that are in decline for part of their history.

## Methods

### Simulating clade diversification

**Fig. 3:**
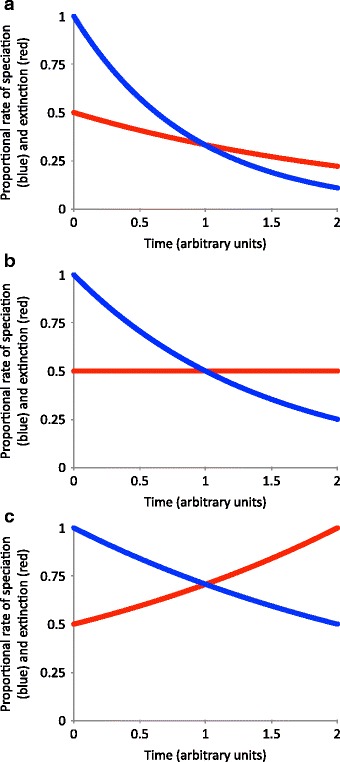
Graphical depiction of speciation and extinction simulation. The blue line represents λ, and the red line represents μ (see Table 5). **a** λ and μ begin high, then decay exponentially through time, with the slower rate of decay in μ eventually leading to extinction outpacing λ. **b** λ begins high and decays exponentially, while μ remains constant. **c** λ begins high and decays, while μ begins low and increases. λ is scaled to 1 and μ to 0.5, representing that our simulations had an initial μ rate half that of λ. For trials in which the λ_1_ was greater than λ_0_, the multiplier for the derived state was in addition to that shown here; thus, λ_1_ began at 1.05 or 1.33 in these simulations, rather than 1. Time is scaled such that one unit represents approximately the amount of time necessary for the two rates to reach equal levels; before one time unit has passed, a clade is still diversifying, but afterwards, it is declining. For each set of state parameters, we ran 100 trials with each that ending at time *t* = 0.25, 0.5, 0.75, 1, 1.5, and 2. In real-world taxa, the exact length of time that one unit corresponds to, as well as the relative decay constants, varies among groups [10]

To do these analyses we conducted birth-death simulations of diversification in which rate of origination (λ) and extinction (μ) change over time (see Table 5). The code for this program is contained in Additional file 1 and was run on a Unix (Mac OSX) operating system. In all of our simulations, λ starts out high and then decays exponentially through time. We ran simulations under a variety of conditions, featuring exponentially decreasing (Fig. 3a), constant (Fig. 3b), or exponentially increasing (Fig. 3c) extinction rates (Table 6). Even in the case of decreasing extinction, the rate at which extinction decays is slower than the rate at which speciation decays, with the result that speciation eventually drops below extinction, and the clade begins to lose diversity.

**Table 5:** List of abbreviations used referring to evolutionary rate parameters

Symbol	Parameter type	Associated character states	“Real” (simulated) or estimated from BiSSE
λ_0_	Speciation rate	Ancestral state	Real
λ_1_	Speciation rate	Derived state	Real
μ_0_	Extinction rate	Ancestral state	Real
μ_1_	Extinction rate	Derived state	Real
q01	Transition rate	Ancestral to derived	Real
q10	Transition rate	Derived to ancestral	Real
$\widehat{\uplambda}$_0_	Speciation rate	Ancestral state	Estimated from BiSSE
$\widehat{\uplambda}$_1_	Speciation rate	Derived state	Estimated from BiSSE
$\widehat{\upmu}$_0_	Extinction rate	Ancestral state	Estimated from BiSSE
$\widehat{\upmu}$_1_	Extinction rate	Derived state	Estimated from BiSSE
$\widehat{\mathrm{q}}$01	Transition rate	Ancestral to derived	Estimated from BiSSE
$\widehat{\mathrm{q}}$10	Transition rate	Derived to ancestral	Estimated from BiSSE

Parameters with the subscript 0 refer to rates associated with the ancestral state, those with the subscript 1 refer to the derived state, parameters with the hat symbol (^) are estimates made by BiSSE, while parameters with no hat symbol are the real values in the simulation

**Table 6:** Schematic of the data analyses. Test type refers to whether the simulation was run to test for the propensity of BiSSE to indicate a most likely model with higher diversification rates in the ancestral state when the character has no effect (“asymmetry bias”) or to test the propensity of BiSSE to indicate a most likely model with higher diversification rates in the derived state when the character does have an effect (“Asymmetry detection”).

Test type	Morphologic evolution model	λ ratio	Extinction rate change model
Asymmetry bias	Test to determine if the best BiSSE model has evolutionary rates depending on character states (i.e. $\widehat{\uplambda}$_0_ ≠ $\widehat{\uplambda}$_1_, when λ_0_ = λ_1_).		
Asymmetry bias	Punctuated	1.0	Decreasing
Asymmetry bias	Continuous-time	1.0	Decreasing
Asymmetry bias	Continuous-time	1.0	Constant
Asymmetry bias	Continuous-time	1.0	Increasing
Asymmetry detection	Test to determine if BiSSE correctly models evolutionary rates to depend on character states (i.e. $\widehat{\uplambda}$_0_ = $\widehat{\uplambda}$_1_, when λ_0_ = λ_1_).		
Asymmetry detection	Punctuated	1.33	Decreasing
Asymmetry detection	Punctuated	1.05	Decreasing
Asymmetry detection	Continuous-time	1.33	Decreasing

Morphological evolution model refers to whether changes in character states were assumed to happen only during speciation events (“Punctuated”) or whether character states could evolve at any point in a species’ history, being independent of speciation events (“Continuous-time”). λ ratio signifies the ratio of λ_1_/λ_0_. The extinction rate change model represents what simulated long term trends existed in μ (in all runs, speciation rate was decreasing, and more rapidly than extinction). All combinations of models were run with six different simulation lengths of 0.25, 0.5, 0.75, 1.0, 1.5, and 2.0, where length 1 is the time required for speciation rate to decay to the point where speciation and extinction rates are equal. Each combination of parameters (including length of simulation) was run 100 times. Abbreviations of λ and μ are explained in Table 5

The simulated phylogenetic trees were analyzed with BiSSE, which takes a tree with branch lengths and character states mapped onto the tips and fits models across the tree using maximum likelihood [2]. Because both our computer simulations and BiSSE are model-based, we make the following distinction for the sake of clarity: in this paper, we use the word “simulation” to refer to the simulated phylogeny incorporating different diversification parameters, the word “model” to refer to likelihood models fit using the BiSSE method, and the word “run” to represent an individual experiment from simulation to model-fitting. BiSSE models have six parameters: rate of origination (λ), extinction (μ), and state transition (q), each of which have values for derived (1) and ancestral (0) character states (Table 5). We use the term “neutral” to describe simulated characters that do not affect evolutionary rates in any way (i.e. λ_0_ = λ_1_, μ_0_ = μ_1_, q01 = q10). We likewise use the term “non-neutral” to describe a character that is not neutral by the above definition.

We conducted two sets of analyses, the first to determine if the BiSSE model with the highest likelihood includes a non-neutral character when the simulated character is neutral (e.g., λ_0_ = λ_1_ in the simulation, but the most likely parameter estimates from the BiSSE analyses have $\widehat{\uplambda}$_0_ ≠ $\widehat{\uplambda}$_1_, and likewise for μ and q). The second group of runs was to determine if declining speciation rate results in the most likely BiSSE model having the character being neutral when the character in the simulation is non-neutral (i.e. λ_0_ ≠ λ_1_, but $\widehat{\uplambda}$_0_ = $\widehat{\uplambda}$_1_). For this second set of analyses, we simulated two different ratios of λ_1_ to λ_0_, one in which λ_1_ = 1.33 x λ_0_, and one in which λ_1_ = 1.05 x λ_0._

We began all simulations with extinction rate at half the speciation rate, and defined a single time unit as the time required for speciation and extinction rate to become equal (the point of peak richness). For real clades in the fossil record, the number of years to peak richness varies widely [10]. In our shortest runs (ended at 0.25 time units), speciation rate still well exceeds extinction rate and the clade is rapidly diversifying. At the end of the longest (two time units) simulations, speciation rate is half that of extinction rate and the clade is declining.

Our speciation rate asymmetries between λ_0_ and λ_1_ for non-neutral characters are smaller than those that Davis et al. [25] investigated. We did this for the following reason: at time 0, λ_0_ is twice μ_0_. At time 2, λ_0_ is half μ_0_. Because λ_1_ is a flat multiple of λ_0_, increasing the ratio of the two rates to 2:1 or beyond would result in species with the derived state continuing to have net positive diversification rates even at the end of our longest runs. We therefore used smaller, but still paleontologically realistic (e.g. [10]) values of 1.33 and 1.05 as the ratio of λ_1_ to λ_0_. Additionally, this real rate asymmetry lies in the opposite direction (i.e. λ_1_ > λ_0_) from what we predicted the most likely BiSSE model to be ($\widehat{\uplambda}$_0_ > $\widehat{\uplambda}$_1_). We did this because we hoped that the direction of the rate asymmetry in the best model would enable us to distinguish between a model-fitting bias created by our simulations’ violation of BiSSE’s assumptions as opposed to detection of real asymmetry created by our simulated non-neutral character.

**Fig. 4:**
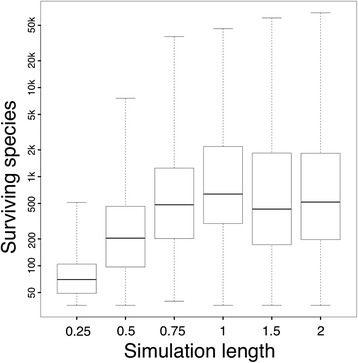
Number of species surviving to end of simulation as a function of length of our simulation. The dark lines are the median number of surviving species, the boxes the first and third quartiles, and the whiskers the maximum and minimum

We ran 100 simulations with each combination of parameters. Simulations that had fewer than 50 surviving species were re-run until the simulation had a total of 50 surviving species (36 for our punctuated equilibrium simulation – see below). Thus, each combination of parameters had a total of 100 simulations for which there was at least the minimum quota of species, and in most of our runs the number of surviving species is considerably larger (Fig. 4). We excluded simulation runs with few surviving species because Davis et al. [25] demonstrated that the statistical power to distinguish among BiSSE models is reduced when examining small clades. By setting this arbitrary cutoff, we introduce a significant acquisition bias to our study. This bias also exists in real clades, however, as workers tend to analyze large clades and especially those that have an imbalance in richness among species bearing specific traits. Because this acquisition bias exists in analyses of real clades, we decided not to correct for it, as doing so would have been both methodologically difficult and would introduce yet additional biases.

### Simulating morphological evolution

We ran two types of simulation of character evolution in the context of our declining speciation and extinction rates. The first (“continuous-time”) type of simulation allows the character state of a species to change at any time, not just at speciation events, and thus emulates anagenetic character evolution. We set the values of q01 and q10 to 0.6 changes per time unit. This number matches the state transition rates typical of the punctuated equilibrium simulation described below. In the continuous simulation, q10 and q01 are time-invariant, irrespective of how λ and μ may be changing. The expected number of changes along the line of any surviving species from the root of the tree to the tip is thus the same regardless of how many branching events took place. This simulation is implicit in the assumptions of BiSSE as implemented in diversitree [26], and is equivalent to “phyletic gradualism” sensu Eldredge and Gould [18].

The second (“punctuated”) type of simulation emulates cladogenetic character evolution in which state changes happen only at speciation events. As a consequence, q01 and λ_0_ are linked and change together, as are q10 and λ_1_. We used a probability of state change (in either direction) of 0.09 per branching event, which is typical of minimum steps parsimony-based phylogenetic reconstructions of fossil taxa [27]. The expected number of total state changes along any branch thus depends on the number of branching events, and is independent of time required for those branching events to occur. The shortest simulations yield a geometric mean of 4.7 changes and the longest 15.2 changes, but because of extinction, not all of these changes appear in terminal taxa. This punctuated equilibrium simulation represents yet another violation of BiSSE’s assumptions in addition to time-variant speciation and extinction rates. Newer models, such as ClaSSE [19] do allow for punctuated equilibrium scenarios. However, we chose to use BiSSE because we sought to quantify whether anagenetic and cladogenetic forms of character evolution impact the biases that we here investigate.

### Assessment

We conducted BiSSE analyses of our simulated clades using the diversitree package in R [26]. BiSSE models in diversitree are compared using likelihood ratio tests in the ANOVA function in R to assess the significance level of asymmetries between $\widehat{\uplambda}$_0_ versus $\widehat{\uplambda}$_1_, $\widehat{\upmu}$_0_ versus $\widehat{\upmu}$_1_, and $\widehat{\mathrm{q}}$01 versus $\widehat{\mathrm{q}}$10. The null hypothesis of these likelihood ratio tests is that the character is neutral (i.e. $\widehat{\uplambda}$_0_ = $\widehat{\uplambda}$_1_, $\widehat{\upmu}$_0_ = $\widehat{\upmu}$_1_, $\widehat{\mathrm{q}}$01 = $\widehat{\mathrm{q}}$10). This is different from the null hypothesis of BiSSE itself, which is that there is no variation in evolutionary rates across the phylogenetic tree, which we violate already by the time-variance of speciation and extinction rates in our simulation. Thus, for the sake of clarity, we do not discuss these errors as type-I and type-II in this paper. Because of our continually decreasing speciation rates, there is no BiSSE model that correctly represents our simulated phylogeny, but some BiSSE models are more incorrect than others. We assessed the statistical significance of the frequency by which the best BiSSE model included a neutral or non-neutral character using these likelihood ratio tests (e.g., if λ_0_ = λ_1_, how often does AIC indicate that $\widehat{\uplambda}$_0_ = $\widehat{\uplambda}$_1_ is the best model and how often does AIC imply that $\widehat{\uplambda}$_0_ ≠ $\widehat{\uplambda}$_1_ is a better model?).

## Additional file

Additional file 1: Speciation extinction simulation model. Source code (in C) for the simulation program used to generate the data used in this study. This file is also available via Dryad. (ZIP 12 kb)

## Abbreviations

BiSSE: Binary state speciation and extinction
MuSSE: Multiple state speciation and extinction
QuaSSE: Quantitative state speciation and extinction
